# Mechanisms of Bacterial Resistance and Innovative Strategies to Overcome Antimicrobial Resistance

**DOI:** 10.3390/antibiotics15030319

**Published:** 2026-03-20

**Authors:** Irene Dini

**Affiliations:** Department of Pharmacy, University of Naples Federico II, 80131 Napoli, Italy; irdini@unina.it

**Keywords:** antibiotic resistance history, resistance mechanisms, multidrug resistance mechanisms, next generation of antibiotics, monoclonal antibodies, nano formulations

## Abstract

Widespread, sometimes careless use of antibiotics has accelerated the rise and spread of antibiotic-resistant pathogens. These resistant bacteria are now often found in animal-based foods like meat, milk, and eggs, as well as in plant-based foods such as fruits and vegetables. Contaminated food is a key way these bacteria travel through the food chain and eventually reach people. This review brings together global trends in antibiotic contamination, explains the molecular mechanisms underlying antimicrobial resistance, and examines current approaches to addressing this problem. It also highlights new technologies that could work alongside or improve on traditional antibiotics. Some promising options are antimicrobial peptides, natural bioactive compounds, nanomaterials, and monoclonal antibody-based therapies. Tackling antimicrobial resistance requires teamwork across fields such as microbiology, food science, pharmacology, environmental science, and public health. Future research should strengthen global surveillance, standardize resistance-assessment methods, expand studies on non-bacterial pathogens, and ensure rigorous evaluation of novel therapies for pharmacokinetics, toxicity, scalability, and regulatory compliance. Ongoing global cooperation and new scientific ideas are crucial to slow the spread of resistant microbes and protect food safety and human health.

## 1. Introduction

Antibiotics are natural (e.g., β-lactams produced by *Actinomycetes* and aminoglycosides produced by *Streptomyces* spp.) or synthetic (e.g., sulfonamides, oxazolidinones) substances that kill bacteria or inhibit their growth [1]. Their use dates back to antiquity.

The ingestion of tetracycline has been documented in Nubian (350–550 CE) and late Roman-period remains. In contrast, ancient Egyptians appear to have applied tetracycline topically, as suggested by moldy bread placed on wounds, an early empirical antimicrobial practice [2,3,4]. The term “antibiotics” was coined following Selman Waksman’s pioneering work, which led to the isolation of microbial compounds capable of inhibiting the growth of other microorganisms [5]. The contemporary notion of antibiotics can be traced back to Paul Ehrlich’s ‘magic bullet’ concept, which envisioned agents capable of selectively eliminating pathogenic microorganisms while leaving host tissues unharmed [6], the development of Salvarsan for syphilis [7], and Fleming’s discovery of penicillin in 1928 [8]. The identification of numerous antibiotic classes still in clinical use occurred during the post-World War II era. The antibiotics streptomycin, chloramphenicol, tetracyclines, erythromycin, vancomycin, and cephalosporins transformed once-lethal infections into treatable conditions [9]. Innovation slowed from the 1960s onward due to declining pharmaceutical investment, leading to a long gap in the development of new broad-spectrum agents. Efforts focused on modifying existing chemical scaffolds rather than pursuing entirely new classes [10]. The synthesis of amoxicillin and quinolones marked a significant advancement, offering improved stability and broader antimicrobial spectra. Vancomycin emerged as a key agent against methicillin-resistant *Staphylococcus aureus* (MRSA), while newer antibiotics, including macrolides, third-generation cephalosporins, daptomycin, and linezolid, were developed to combat Gram-negative pathogens and enhance pharmacokinetic profiles [11]. Antibiotic actions can involve blocking the synthesis of bacterial cell walls (e.g., glycopeptide and β-lactam antibiotics), damaging membrane integrity (e.g., polymyxins and cyclic lipopeptides), inhibiting the production of nucleic acids (e.g., rifamycins and quinolones) and proteins (e.g., aminoglycosides, tetracyclines, macrolides, streptogramin B, lacosamides, and oxazolidinones), and interfering with vital metabolic functions (e.g., sulfonamides alone or co-formulated with trimethoprim) [9]. Today, the extensive use of antibiotics in medicine, aquaculture, and agriculture has created significant environmental and public health challenges. These sectors release substantial amounts of unmetabolized antibiotics into wastewater, surface runoff, and effluents, creating continuous pathways for these compounds to enter natural ecosystems. Once introduced into the environment, antibiotics can destabilize microbial communities and interfere with key ecological processes, including carbon and nitrogen cycling. Such disturbances alter ecosystem functioning and create selective pressures that directly promote the emergence and persistence of antibiotic-resistant bacteria (ARB) and antibiotic resistance genes (ARGs) [12]. Municipal wastewater, livestock farms, and hospital waste are the primary sources and routes for ARGs, which help spread multidrug-resistant organisms and reduce the effectiveness of current treatments [13]. Microbiomes across environmental compartments, including soil, water, and sediments, serve as major reservoirs for ARB and ARGs, where they can accumulate, evolve, and disseminate. ARG profiles differ by region and environmental source, creating specific ecological risks and complicating monitoring [14]. The human gut microbiome is another key reservoir that can support the growth and spread of ARGs and ARBs. Overuse or misuse of antibiotics can upset the balance of gut microbes, leading to dysbiosis and increasing the risk of metabolic, inflammatory, and immune diseases. It also accelerates the selection and spread of ARB and ARGs, contributing to the global rise in antimicrobial resistance (AMR) [15]. AMR causes more than a quarter of hospital-acquired infections and is a serious global health issue. Tools such as the Nemerov multi-factor index, probability density function models, and ecological risk assessment frameworks can be used to measure resistance levels. If the problem is not addressed, infections from ARB could lead to as many as 10 million deaths each year by 2050 [16].

This review examines the historical development of antibiotics, the molecular and ecological drivers of resistance, and emerging therapeutic strategies to help control the spread of resistant bacteria. By integrating environmental, microbiological, and pharmacological perspectives, the review aims to provide a comprehensive framework for understanding current challenges and guiding future innovation in the fight against AMR.

## 2. Antibiotic Resistance

**Figure 1:**
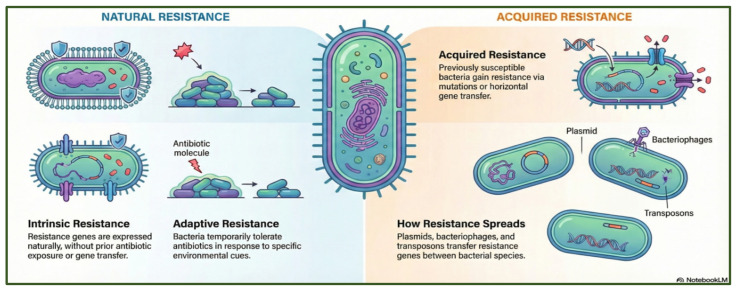
Overview of natural and acquired antibiotic resistance in bacteria, highlighting intrinsic and adaptive tolerance mechanisms, as well as mutation-driven and horizontally transferred resistance spread via plasmids, bacteriophages, and transposons. The figure was generated using NotebookLM (Gemini 1.5 model), consulted in February 2026.

Antibiotic resistance is a multifaceted phenomenon driven by the extensive use of antibiotics across the clinical, agricultural, and food production sectors, as well as by microbial population dynamics and environmental pressures [17]. Relevant resistance mechanisms include intrinsic, acquired, and adaptive pathways (Figure 1).

Intrinsic resistance is linked to inherent structural features such as reduced membrane permeability, porin remodeling, or modifications of glycopeptide targets [18,19]. Additional mechanisms, including lipid A modification via the *pmrCAB* operon, have been described in *Proteus mirabilis*, *Serratia marcescens*, *Burkholderia* spp., and *Yersinia* spp. [20,21].

Acquired resistance arises through spontaneous mutations or horizontal gene transfer (HGT), mediated by plasmids, bacteriophages, transposons, and integrons [22]. These mobile genetic elements accelerate the dissemination of resistance determinants across species and environments.

Plasmids are pieces of DNA outside the chromosome that replicate and transfer resistance genes directly between cells via a sex pilus in both Gram-negative and Gram-positive bacteria. They often carry several resistance genes, helping multidrug resistance spread quickly [23].

Bacteriophages facilitate HGT by packaging and delivering bacterial DNA fragments into other cells during infection [24].

Transposons, sometimes called ‘junk DNA,’ are mobile genetic elements that do not encode proteins but can relocate within the genome via distinct mechanisms. Type I elements (retrotransposons) employ an RNA intermediate in a “copy-and-paste” process, whereas Type II elements (DNA transposons) move via a “cut-and-paste” mechanism. Both types are grouped into clades based on their sequences and whether they have their own transposition machinery [25].

Integrons represent another platform for resistance; they consist of an integrase gene (*intI*), a recombination site (*attI*), and one or more gene cassettes. Class 1 integrons, particularly prevalent in Gram-negative bacteria, are strongly associated with multidrug resistance [26].

Adaptive resistance, in contrast, is a transient and reversible state that enables microorganisms to withstand antibiotic exposure in response to specific environmental cues. Contributing factors include altered expression of resistance-related genes, restricted antibiotic penetration within biofilms, and reduced metabolic activity in deeper biofilm layers. Environmental stresses, such as nutrient limitation, pH shifts, ion fluctuations, changes in growth phase, or exposure to subinhibitory antibiotic concentrations, can also induce this phenotype [27]. Unlike intrinsic or acquired resistance, adaptive resistance dissipates once the triggering conditions are removed, restoring bacterial susceptibility to antibiotics [28].

**Figure 2:**
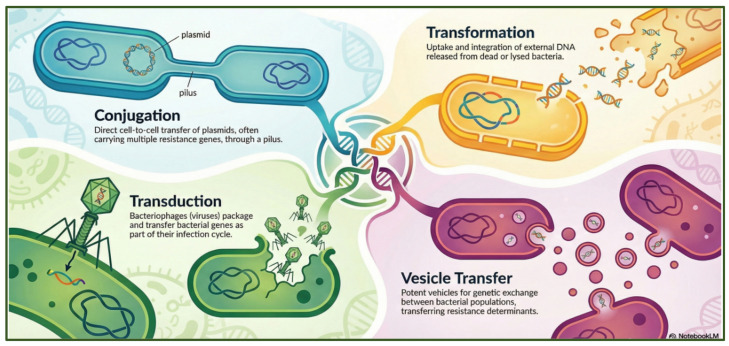
Mechanisms of horizontal gene transfer in bacteria that drive the dissemination of antimicrobial resistance genes. The figure was generated using NotebookLM (Gemini 1.5 model), consulted in February 2026.

Antibiotic-resistant pathogens spread resistance genes using several horizontal gene transfer (HGT) methods, including conjugation, transformation, transduction, and membrane vesicles (Figure 2).

Conjugation involves direct cell-to-cell contact, typically mediated by conjugative plasmids or integrative and conjugative elements, which transfer DNA via a pilus or mating bridge, enabling rapid dissemination of multidrug-resistance determinants within and between species. Transformation occurs when naturally competent bacteria take up free extracellular DNA released from lysed cells in the environment and stably integrate resistance genes into their chromosomes or plasmids, thereby expanding their adaptive potential. Transduction is mediated by bacteriophages, which inadvertently package bacterial DNA, including antimicrobial resistance genes, during their replication cycle and deliver it to new bacterial hosts upon infection, linking viral dynamics to the spread of AMR. In addition, many bacteria release membrane vesicles, nano-sized lipid bilayer particles that encapsulate DNA, RNA, and proteins; these vesicles protect genetic material from degradation and can fuse with recipient cells, delivering resistance genes even across species or genus boundaries [29].

### 2.1. Antibiotic Resistance in Clinical and Medical Settings

**Figure 3:**
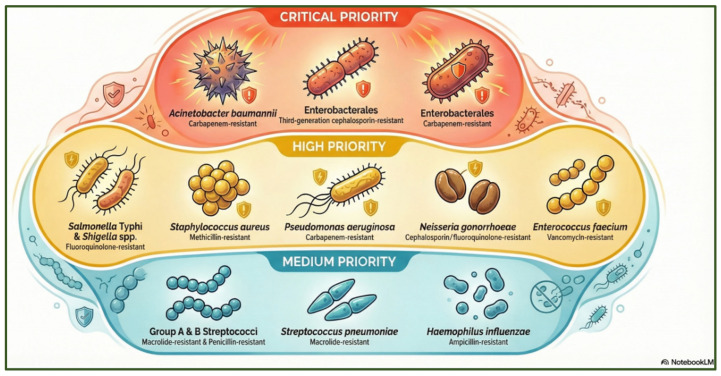
WHO priority categories of antibiotic-resistant bacterial pathogens, highlighting critical, high, and medium-priority species associated with major antimicrobial resistance threats. The figure was generated using NotebookLM (Gemini 1.5 model), consulted in February 2026.

The clinical sector has historically been the first arena in which antibiotic resistance emerged and was documented. Shortly after the introduction of penicillin, Abraham and Chain (1940) reported penicillinase-producing *Escherichia coli*, marking the earliest known enzymatic degradation of an antibiotic [30]. Penicillin-resistant strains were identified in 1942 [31]. Methicillin-resistant *Staphylococcus aureus* (MRSA) emerged in the United Kingdom in 1962 [32]. Recent EU surveillance (2019–2020) reports high resistance in *Salmonella* spp. to sulfonamides, ampicillin, and tetracyclines (50–80%), while resistance to third-generation cephalosporins remains low (0.5–0.8%) [33]. Between 2016 and 2020, overall resistance reached 42.9% in Italy and 35% in Belgium [34]. To address this emerging challenge and guide global priorities, the World Health Organization (WHO) has established a list of critical, high-, and medium-priority resistant pathogens based on epidemiological and clinical criteria (Figure 3) [35,36].

### 2.2. Antibiotic Resistance in Agriculture and Aquaculture

Agriculture and aquaculture represent major drivers of global antibiotic consumption. Current estimates indicate that antibiotic use in animal production amounts to 63,151 ± 1560 tons, with projections suggesting a 67% increase to 105,596 ± 3605 tons by 2030; Asia alone is expected to account for 46% (51,851 tons) of this total [37]. More than half of the antibiotics used in livestock belong to classes also employed in human medicine, creating cross-sectoral selective pressures that facilitate the emergence and spread of resistance [38]. The widespread availability of antimicrobials through farm supply chains often leads to unregulated or excessive use, particularly in intensive farming systems. Wastewater, manure, and runoff from livestock and aquaculture operations serve as major environmental reservoirs of ARB and RGs, promoting their dissemination into soil, water, and surrounding ecosystems [10]. Environmental contaminants further exacerbate resistance dynamics. Microplastics, for example, provide persistent surfaces that support biofilm formation, enhance microbial protection, and facilitate horizontal gene transfer. Their accumulation across water, soil, and food webs makes them long-term reservoirs for ARB and ARGs [38,39].

### 2.3. Antibiotic Resistance in the Food Production Chain

In food production, contamination can occur at multiple points along the farm-to-fork continuum, and foods of animal origin represent the primary route by which ARB enter the human food chain. Resistant *Salmonella*, *Campylobacter*, and *Escherichia coli* are frequently associated with poultry, eggs, pork, beef, and turkey products, due to the high selective pressures generated by intensive farming practices [40,41]. Although surveillance data for foods of non-animal origin are limited, outbreaks between 2007 and 2011 indicate that plant-derived foods accounted for approximately 10% of foodborne pathogen events, with leafy greens, tomatoes, melons, legumes, and grains implicated in the spread of AMR *Salmonella* and *Escherichia coli* [42]. Additional routes include food handlers, whose poor hygiene can facilitate cross-contamination, and sublethal food-processing technologies that may alter AMR phenotypes or promote gene transfer [43].

## 3. Genetic and Biochemical Bases of Bacterial Resistance to Antibiotics

**Figure 4:**
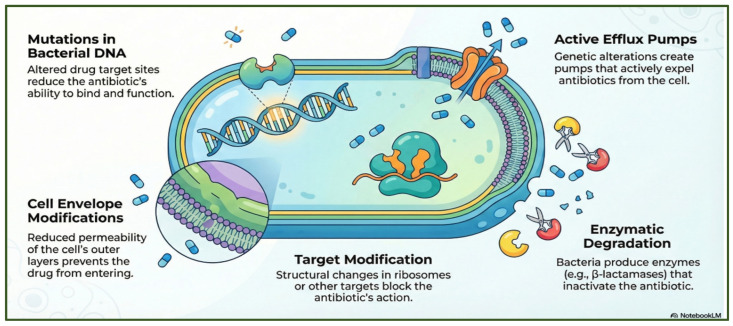
Key bacterial mechanisms of antibiotic resistance. The figure was generated using NotebookLM (Gemini 1.5 model), consulted in February 2026.

Bacteria employ a wide range of molecular strategies to evade the effects of antibiotics. These include genetic mutations or insertions that alter the transcriptional regulation of resistance determinants; overexpression of efflux pumps that expel diverse antimicrobial agents; reduced membrane permeability that limits antibiotic uptake [44]; enzymatic degradation or chemical modification of antibiotic molecules [45]; and structural alterations of target sites that decrease drug-binding affinity [46] (Figure 4).

### 3.1. Mutations in Bacterial DNA

Random mutations in bacterial DNA can modify gene sequences, and even a single nucleotide change may alter an amino acid residue. Such alterations can influence enzyme activity or the function of essential cellular components, ultimately contributing to antibiotic resistance [47]. Mutations can change drug targets or metabolic pathways, and bacteria can also share resistance genes through horizontal gene transfer. Mutations may affect drug targets or disrupt metabolic pathways, while horizontal gene transfer enables bacteria to disseminate resistance determinants across populations. As a result, key resistance genes, including *npmA*, *rmtA*, *bla*, *cat*, *msr*, and *erm*, often become upregulated, conferring resistance to agents such as rifampicin, fusidic acid, streptomycin, fluoroquinolones, and oxazolidinones [48]. Sulfonamides exert their antimicrobial activity by inhibiting dihydropteroate synthase (DHPS), a pivotal enzyme in folate biosynthesis. Their structural similarity to para-aminobenzoic acid (PABA) allows them to inhibit DHPS, thereby competitively disrupting nucleotide synthesis. Resistance commonly arises through mutations in the *folP* gene or through acquisition of plasmid-encoded *sul* genes (*sul1*–*sul4*), which generate DHPS variants with reduced affinity for the drug [47]. Fosfomycin blocks the MurA enzyme, which is needed to make peptidoglycan, by attaching to the Cys115 site. Substitution of Cys115 with Asp disrupts this interaction and diminishes drug efficacy. Additional resistance mechanisms involve mutations in the GlpT and UhpT transporters, which mediate fosfomycin uptake; impaired transport reduces intracellular drug accumulation and further enhances resistance [49]. Ciprofloxacin exerts bactericidal activity by inhibiting DNA gyrase, preventing the relaxation of supercoiled DNA, and inducing double-strand breaks. In *Escherichia coli*, exposure to ciprofloxacin triggers pronounced cell elongation and increased chromosomal replication, activating the SOS response. This DNA repair pathway elevates mutation rates and accelerates the emergence of multidrug resistance within a single bacterial cell [50].

### 3.2. Genetic Alterations in Efflux Systems

Efflux pumps (EPs) are membrane-embedded transport proteins that expel toxic compounds, including antibiotics, from bacterial cells, thereby playing a central role in multidrug resistance. Beyond their function in drug extrusion, EPs contribute to virulence, biofilm development, and overall pathogenicity. These systems may be encoded on chromosomal DNA or plasmids and exhibit broad or narrow substrate specificity, enabling the export of antibiotics, detergents, dyes, and various metabolic byproducts. Advances in molecular microbiology have facilitated the identification of numerous efflux pumps in clinically significant pathogens such as *Klebsiella pneumoniae*, *Staphylococcus aureus* (MRSA), *Streptococcus pneumoniae*, *Listeria monocytogenes*, *Pseudomonas aeruginosa*, *Acinetobacter baumannii*, *Escherichia coli*, *Enterococcus* spp., *Salmonella* spp., and *Campylobacter jejuni* [51]. These transporters are classified into six major families: MATE (Multidrug and Toxic Compound Extrusion), PACE (Proteobacterial Antimicrobial Compound Efflux), RND (Resistance-Nodulation-Division), SMR (Small Multidrug Resistance), ABC (ATP-Binding Cassette), and MFS (Major Facilitator Superfamily) [52]. MFS, PACE, and SMR pumps function as secondary active transporters that rely on the proton motive force (PMF) to couple proton influx with the efflux of antibiotics or other toxic molecules. MATE transporters utilize either a proton gradient or a sodium ion gradient, depending on the organism and environmental conditions. In contrast, the ABC superfamily uses ATP hydrolysis to drive substrate export independently of ion gradients [53]. RND pumps, found only in Gram-negative bacteria, form tripartite systems that span both membranes. Examples include AcrAB-TolC (*Escherichia coli*), AdeABC (*Acinetobacter baumannii*), and MexAB-OprM (*Pseudomonas aeruginosa*). In Gram-positive bacteria, MFS pumps like NorA in *Staphylococcus aureus* are responsible for fluoroquinolone resistance.

Efflux pump inhibitors (EPIs), derived from natural products and synthetic compounds, offer a promising approach to combat multidrug resistance. Many studies have shown that using EPIs with antibiotics blocks efflux-mediated drug removal and restores antimicrobial activity. This strategy not only resensitizes resistant bacteria but also reduces the likelihood of selecting resistant mutants [54]. EPIs can be divided into competitive and non-competitive substrate inhibitors. Competitive EPIs, which also serve as substrates for the transporters they target, may inadvertently trigger efflux pump overexpression, thereby diminishing their therapeutic effectiveness. In Gram-negative bacteria, the outer membrane serves as a significant permeability barrier, restricting antibiotic entry. Nonetheless, certain EPIs, such as PAβN and polyamino-isoprene derivatives, can cross or circumvent this barrier, promoting intracellular antibiotic accumulation and restoring antimicrobial activity [55]. Other EPIs act by increasing membrane permeability through direct membrane disruption; however, this mechanism carries the risk of off-target toxicity and unintended bacterial cell lysis [56]. Therefore, EPIs capable of entering bacterial cells without disrupting membrane proteins are particularly desirable, as they minimize off-target toxicity and offer greater therapeutic potential for overcoming multidrug resistance.

### 3.3. Cell Envelope Modifications

Gram-positive and Gram-negative bacteria exhibit distinct cell wall architectures. Gram-negative bacteria possess a tripartite envelope consisting of an outer membrane enriched with lipopolysaccharide, a thin peptidoglycan layer, and an inner asymmetric cytoplasmic membrane composed of phospholipids on the inner leaflet and lipopolysaccharides on the outer leaflet [57]. Peptidoglycan is composed of glycan chains of N-acetylglucosamine and N-acetylmuramic acid, cross-linked via transpeptidation catalyzed by penicillin-binding proteins (PBPs), including transpeptidases and carboxypeptidases. β-lactams, glycopeptides, and fosfomycin disrupt bacterial cell wall integrity, leading to bacterial death [58]. β-lactam antibiotics, including penicillins, carbapenems, cephalosporins, and monobactams, induce bacterial cell lysis by inhibiting penicillin-binding proteins, enzymes with transpeptidase activity. PBPs catalyze the cleavage of the terminal D-alanine from one stem peptide and subsequently form covalent cross-links with adjacent peptide chains, generating the rigid, mesh-like peptidoglycan structure that maintains cell wall integrity. The bactericidal effect of β-lactams ultimately depends on the activity of autolysins, such as L-alanyl N-acetylmuramic acid amidase, collectively known as murein hydrolases. These enzymes degrade peptidoglycan by hydrolyzing specific bonds, thereby weakening the cell wall and leading to lysis. However, mutant strains with reduced or defective autolysin activity can evade this lytic response. Although β-lactams still inhibit their growth, these mutants avoid cell rupture and display a phenotype known as penicillin tolerance [59]. Glycopeptides (i.e., vancomycin, teicoplanin, dalbavancin, oritavancin, telavancin, or corbomycin) block transglycosylation and transpeptidation, thereby interrupting peptidoglycan assembly and inducing bacterial lysis. They achieve this by binding to the D-Ala D-Ala termini of peptidoglycan precursors. Their activity is particularly potent against Gram-positive bacteria, including methicillin-resistant *Staphylococcus aureus* (MRSA), because they cannot penetrate the outer membrane of Gram-negative bacteria [60]. Glycopeptide resistance stems from the replacement of D-Ala-D-Ala in peptidoglycan precursors with D-Ala-D-Lac or D-Ala-D-Ser, which bind vancomycin poorly. Resistance to glycopeptides arises when bacteria replace the D-Ala-D-Ala motif with D-Ala-D-Lac or D-Ala-D-Ser in their cell wall precursors, significantly reducing vancomycin binding affinity. The vanRSHAX gene cluster mediates this structural modification (first identified in livestock-associated strains) [61]. Fosfomycin inhibits early steps of peptidoglycan biosynthesis by targeting MurA, an essential enzyme in cell wall formation. In Gram-positive bacteria, resistance may result from structural alterations in MurA, the production of β-lactamase or FosA enzymes that inactivate the drug, and reduced membrane permeability that limits intracellular accumulation [49].

### 3.4. Enzymatic Modification

Enzymatic modification refers to resistance mechanisms in which specialized bacterial enzymes chemically alter antibiotic molecules, thereby neutralizing their activity. This strategy plays a significant role in the development of multidrug resistance among clinically significant pathogens, including *Escherichia coli*, *Klebsiella pneumoniae*, *Pseudomonas aeruginosa*, and *Acinetobacter baumannii*. These enzymes may acetylate, phosphorylate, adenylate, or otherwise modify antimicrobial agents, preventing them from binding to their targets and enabling bacteria to survive therapeutic concentrations [62]. Aminoglycoside resistance can arise through enzymatic drug modification mediated by aminoglycoside-modifying enzymes, including kinases, acetyltransferases, and nucleotidyltransferases. In addition, rRNA methyltransferases confer broad resistance by methylating specific residues in 16S or 23S rRNA, thereby blocking the binding of multiple ribosome-targeting antibiotics, such as lincosamides, aminoglycosides, streptogramins, oxazolidinones, and macrolides [61]. β-Lactam resistance frequently involves enzymes such as β-lactamases, which hydrolyze the β-lactam ring of penicillins and cephalosporins, rendering them inactive. Some members of the *Enterobacteriaceae* family produce carbapenemases (carbapenem-hydrolyzing enzymes) that degrade last-resort β-lactams and contribute to severe multidrug-resistant phenotypes [63,64]. Acetyltransferases, phosphotransferases, and adenylyltransferases inactivate aminoglycosides by adding acetyl, phosphate, or adenyl groups, respectively, thereby preventing the drugs from binding to their ribosomal targets. Similar enzymatic mechanisms contribute to resistance against macrolide, lincosamide, and streptogramin antibiotics [65]. Chloramphenicol acetyltransferase likewise neutralizes chloramphenicol by acetylating it, thereby abolishing its ability to inhibit protein synthesis [66]. In addition, some bacteria employ oxidoreductases (such as monooxygenases) to modify antibiotics through oxidation or reduction reactions. These enzymes play a key role in resistance to rifamycins and nitrofurans by altering the structure of these drugs and reducing their antimicrobial activity [67].

### 3.5. Target Modification

Antibiotics exert their activity by binding to specific molecular targets within bacterial cells, but mutations can disrupt these interactions, leading to resistance. Spontaneous mutations occur at a frequency of approximately 10^−8^ to 10^−9^ per cell division. Although most mutations are deleterious, those that enhance bacterial survival under antibiotic pressure can rapidly disseminate through vertical inheritance and horizontal gene transfer, accelerating the spread of resistance within microbial populations [68].

In *Mycobacterium tuberculosis*, rifampicin resistance commonly arises from mutations in the *rpoB* gene, which encodes the β-subunit of RNA polymerase. These mutations alter the drug-binding site, reducing rifampicin affinity and enabling bacterial survival [69,70].

Alterations in penicillin-binding proteins (PBPs) likewise diminish the efficacy of β-lactam antibiotics. In *Staphylococcus aureus*, acquisition of the *mecA* gene (which encodes the low-affinity PBP2a) renders most β-lactams ineffective. Comparable target-modifying mechanisms underlie resistance to other antibiotic classes, including vancomycin, macrolides, lincosamides, and streptogramins, where structural changes in binding sites prevent effective drug–target interactions [71].

## 4. AMR and Its Macroeconomic Implications

As essential antibiotics are progressively removed from treatment guidelines due to rising resistance, infections become increasingly difficult to manage [72]. When first-line therapies fail, clinicians must rely on second- or third-line agents, which are typically more costly, less accessible, and associated with greater toxicity. In many low-income settings, these alternatives are unavailable, forcing reliance on first-line drugs that are rapidly losing effectiveness. According to the WHO’s 2024 report, AMR has become the third leading cause of death globally, responsible for more than one million deaths directly attributable to bacterial AMR and an additional five million deaths indirectly linked to resistant infections [73]. AMR therefore represents not only a critical public health threat but also a profound economic challenge. Analyses by the World Organization for Animal Health and the World Bank indicate that uncontrolled AMR could destabilize healthcare systems and impede global economic growth, particularly in low- and middle-income countries, where illness and mortality from resistant pathogens may reduce workforce capacity and productivity [74]. Within this framework, the agricultural and aquaculture sectors represent critical economic domains where AMR exerts substantial macroeconomic pressure. In livestock production, rising resistance reduces animal health and productivity, increases mortality, and elevates the costs of veterinary care and biosecurity, ultimately diminishing farm profitability and threatening the stability of national food systems [75]. In aquaculture, now one of the fastest-growing food-producing sectors, AMR contributes to treatment failures, mass mortality events, and reduced growth performance, generating significant economic losses and jeopardizing the sustainability of global fish supply chains [75]. Moreover, AMR contamination in aquatic environments can impair ecosystem services, restrict market access due to antimicrobial residue regulations, and undermine international trade competitiveness, amplifying long-term economic vulnerability [76]. AMR-related outbreaks may also discourage travel and place additional strain on public health infrastructures, contributing to broader economic instability. If resistance continues to rise, projections estimate that AMR could cause approximately 38.5 million deaths between 2025 and 2050. Annual healthcare expenditures could reach $325 billion, and global economic losses may exceed $1.7 trillion by 2050. Conversely, timely interventions, such as improving access to effective treatments and investing in the development of new antimicrobials, could yield a return on investment of up to 28:1, potentially saving $960 billion and preventing millions of deaths. In Europe, the burden of antibiotic resistance is already comparable to that of influenza, tuberculosis, and HIV combined [77]. Forecasts consistently show that failing to act on AMR will exacerbate both health and economic pressures. In response, countries have committed to reducing AMR-related mortality by 10% by 2030 through a One Health strategy that integrates human, animal, and environmental health perspectives [78]. Supporting this effort, the European Union has launched a €253 million partnership to advance One Health AMR research and innovation across 30 participating countries [79].

**Figure 5:**
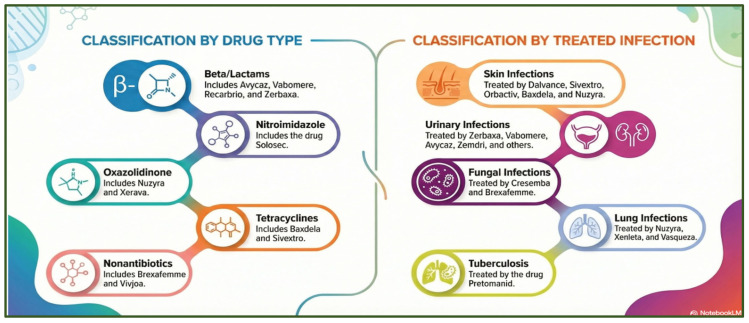
Classification of antimicrobial agents by drug class and by type of infection treated. The figure was generated using NotebookLM (Gemini 1.5 model), consulted in February 2026.

To confront this escalating emergency, the U.S. Food and Drug Administration (FDA) introduced the Qualified Infectious Disease Product (QIDP) designation in 2012 under the Generating Antibiotic Incentives Now (GAIN) Act to incentivize the development of novel antimicrobial agents. This designation applies to drugs intended to treat serious or life-threatening bacterial or fungal infections that are no longer responsive to existing therapies. Products granted QIDP status receive several regulatory advantages, including priority review and an additional five years of market exclusivity [80]. Since the implementation of the GAIN Act, 25 antimicrobials have been approved, 20 of which hold QIDP designation. Between 2013 and 2017, 9 of 13 approved agents received QIDP status, compared with 11 of 12 approvals from 2018 to 2022, a difference that was not statistically significant (69% vs. 92%, *p* = 0.32). Among QIDP-designated products, 9 target genitourinary infections, 6 gastrointestinal infections, 5 skin infections, 3 pulmonary infections, and 1 systemic infection. These include 15 antibacterial agents, 4 antifungals, and 1 antimycobacterial drug [81] (Figure 5).

## 5. Innovative Antimicrobial Approaches

**Figure 6:**
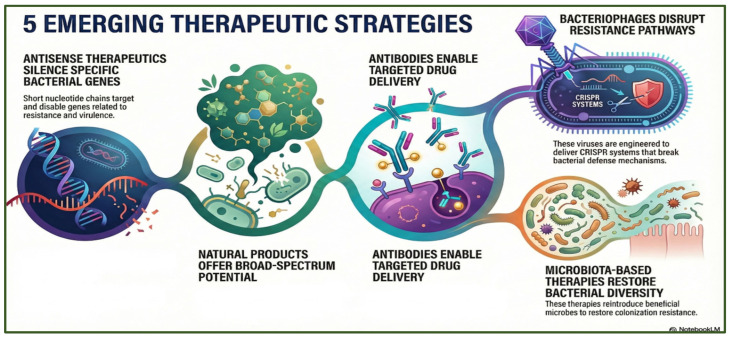
Emerging therapeutic strategies against antimicrobial resistance, including antisense approaches, natural product discovery, antibody-mediated targeting, bacteriophage-based interventions, and microbiota-focused therapies. The figure was generated using NotebookLM (Gemini 1.5 model), consulted in February 2026.

Emerging antimicrobial strategies encompass both indirect, non-growth-inhibitory approaches, such as antivirulence therapies, adjuvants, bacterial consortia, and vaccines (Table 1), and direct approaches, including antisense therapeutics, natural products, antimicrobial peptides (AMPs), bacteriophages, and microbiota-based interventions. These innovative modalities aim to overcome the limitations of traditional antibiotics and offer new avenues for combating multidrug-resistant infections (Figure 6).

### 5.1. Innovative Non-Growth Antimicrobial Approaches

Antivirulence therapeutics exert their activity by interfering with bacterial communication networks such as quorum-sensing pathways, by preventing microbial adherence to host surfaces, or by directly suppressing the expression of specific virulence genes [88].

Quorum sensing is a chemically mediated communication system that enables bacteria to coordinate gene expression in response to population-density signals. Through this mechanism, microbial communities initiate downstream regulatory pathways that modulate adhesion, colonization, and virulence. As cell density increases, quorum-sensing cues drive the shift from an initial pro-adhesive phenotype to the activation of virulence determinants required for infection. This density-dependent regulation ensures synchronized expression of pathogenic traits within the bacterial population [89].

Quorum-quenching approaches are generally grouped into two broad classes. The first includes quorum-quenching enzymes, which act by degrading or inactivating quorum-sensing signal molecules, thereby interrupting the communication network at its source. The second comprises quorum-sensing inhibitors (QSIs), small molecules that chemically disrupt quorum-sensing pathways by competitively blocking the interaction between signal ligands and their cognate receptors [90].

Microbial adherence to host surfaces represents the critical initiating event in the establishment of bacterial colonization. Only after securing a stable attachment can bacteria replicate and subsequently activate virulence programs or transition toward biofilm formation. Interrupting this early adhesive phase, therefore, exerts a pronounced effect [88].

Bacterial appendages, such as flagella and fimbriae, facilitate attachment to biotic and abiotic surfaces; consequently, inhibiting their formation or function represents a viable strategy to prevent microbial adhesion. Anti-adhesion effects can be achieved through biocidal coatings or the application of specific polymers that hinder bacterial cell approach and anchoring to the substrate. In this context, novel polysaccharides derived from Antarctic sponge-associated bacteria and freshwater macroalgae have recently demonstrated the ability to impede bacterial adhesion. Furthermore, advances in nanotechnology have enabled the design of engineered surfaces that intrinsically limit microbial settlement, contributing to the development of next-generation antibacterial materials [91].

Antivirulence drugs constitute an emerging class of therapeutics that act by targeting pathogen-specific virulence factors rather than inhibiting bacterial growth or inducing cell death, thereby functionally disarming infectious agents. Unlike conventional bactericidal antibiotics, which impose strong selective pressures that accelerate the emergence of resistance, antivirulence agents interfere with the molecular interactions between the pathogen and its host. These agents minimize selective pressure by mitigating host damage and attenuating the organism’s capacity to cause disease without killing it [92].

### 5.2. Innovative Direct Antimicrobial Approaches

#### 5.2.1. Antisense Therapy

Antisense therapy employs synthetic antisense oligonucleotides (ASOs) to modulate gene expression by silencing or altering mRNA, thereby inhibiting protein synthesis or modifying splicing patterns. ASOs can induce RNA degradation through RNase H or RNA interference pathways, or they can sterically block translation by obstructing ribosome access or promoting exon inclusion or skipping. Chemical modifications enhance ASO stability, increase binding affinity, and improve cellular delivery, enabling highly targeted therapeutic applications. ASOs have demonstrated the ability to inhibit viral replication, silence pathogenic genes, and modulate immune responses. Owing to their precision and adaptability, they hold considerable promise for personalized medicine [93]. Bacteria represent attractive targets for antisense modulation because transcription and translation occur in the cytoplasm, and endogenous RNases naturally participate in mRNA turnover. Despite strong results in in vitro and in vivo models, no antisense-based therapies have yet entered clinical trials for bacterial infections. Key challenges include expanding the breadth of activity, optimizing cell-penetrating peptide (CPP) designs to enhance intracellular delivery, and evaluating their performance against intracellular bacterial populations [94]. The potential for resistance remains uncertain; however, because ASOs require exact sequence complementarity, even a single nucleotide mutation in the target mRNA could render them ineffective.

#### 5.2.2. Natural Product

**Figure 7:**
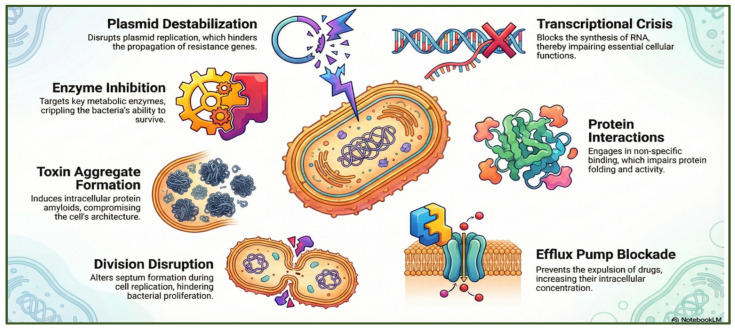
Key antibacterial mechanisms of natural compounds through which bacterial functions are disrupted, including plasmid destabilization, transcriptional inhibition, enzyme blockade, impaired protein interactions, toxin aggregation, division interference, and efflux pump inhibition. The figure was generated using NotebookLM (Gemini 1.5 model), consulted in February 2026.

Plant-derived antimicrobials display extensive chemical diversity and a broad spectrum of biological activities (Figure 7). Their composition varies according to constituent type and concentration, encompassing major antibacterial phytochemicals, including phenols, polyphenols, terpenoids, essential oils, alkaloids, lectins, and polypeptides.

##### Phenols/Polyphenols

**Figure 8:**
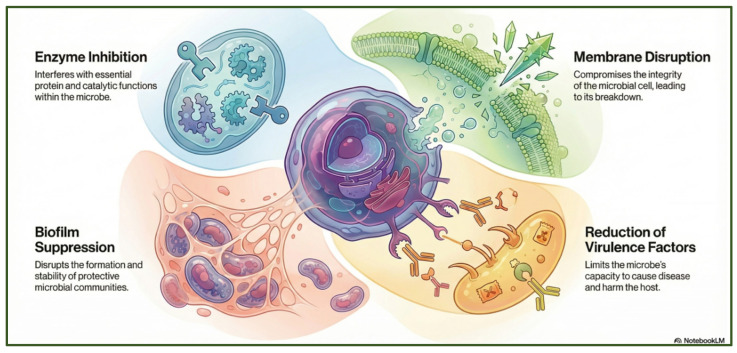
Multitarget antibacterial mechanism of polyphenols. The figure was generated through NotebookLM (Gemini 1.5 model), consulted in February 2026.

Phenols are aromatic compounds containing one or more hydroxyl groups attached to a benzene ring; when multiple hydroxyl groups are present, they are classified as polyphenols [95]. These secondary metabolites are produced by plants and algae [96] and contribute to defense, pigmentation, and ecological interactions [97]. Polyphenols are important in nutrition and medicine because of their bioactive properties [98]. Polyphenols hold significant nutritional and therapeutic value due to their diverse bioactive properties. They exhibit broad-spectrum antibacterial activity, acting against Gram-positive species such as *Staphylococcus aureus* and *Bacillus subtilis*, as well as Gram-negative bacteria such as *Escherichia coli*, *Proteus* sp., *Acinetobacter* sp., *Pseudomonas aeruginosa*, and *Klebsiella pneumoniae* [99]. Polyphenols exert their antimicrobial effects through multiple mechanisms (Figure 8). They can disrupt bacterial cell membranes [100], leading to leakage of essential ions and metabolites and ultimately to cell death [101]. They can also inhibit key metabolic enzymes, thereby interfering with essential biochemical pathways [102]. In addition, polyphenols can prevent biofilm formation or destabilize established biofilms, reducing bacterial adhesion and long-term survival [103]. Some polyphenols further attenuate virulence by suppressing hemolysins, proteases, and other toxins [104].

Flavonoids, a major subclass of polyphenols, can inhibit bacterial electron transport and ATP synthase, thereby impairing energy production [105]. Quercetin-3-O-α-D-arabinofuranoside, identified by D’Agostino et al. in *Alchemilla vulgaris* [106], exhibits reduced lipophilicity, which decreases membrane permeability but enhances solubility and bioavailability, ultimately improving its pharmacological potential [107].

Although neither EFSA nor the FDA has authorized specific antimicrobial claims for phenols or polyphenols in foods or dietary supplements, a substantial body of scientific evidence demonstrates their antimicrobial activity (Table 2).

The total phenolic content of plants can be increased through elicitor treatments, such as the application of *Trichoderma* species and their metabolites [119], and through targeted modulation of phenylpropanoid biosynthesis using genetic and breeding strategies that upregulate key enzymes within polyphenol-producing pathways [120].

Polyphenols may also exert adverse effects, particularly at high concentrations or with prolonged exposure. Their strong redox activity can induce oxidative stress in host tissues, potentially leading to cytotoxicity or genotoxicity [121]. Some phenolic compounds can interfere with drug-metabolizing enzymes, altering the pharmacokinetics of co-administered medications [122]. Gastrointestinal discomfort, asthma, skin irritation, and allergic reactions have also been reported in association with excessive intake of certain polyphenols [123,124]. Therefore, while polyphenols represent promising antimicrobial and antivirulence agents, their safety profiles must be carefully evaluated to ensure appropriate therapeutic use.

##### Betaine

**Figure 9:**
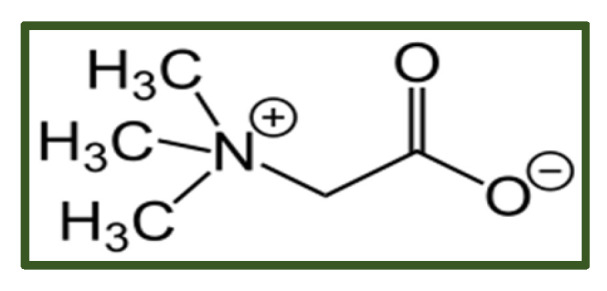
Betaine.

Betaine (trimethylglycine; Figure 9) is a stable, naturally occurring compound present in plants, animals, and microorganisms, with exceptionally high concentrations in spinach, beets, wheat bran, kancolla, wheat germ, and several aquatic invertebrates [125,126,127,128].

Commercial betaine is available in several forms, including natural anhydrous betaine, synthetic anhydrous betaine, and betaine hydrochloride. In the United States, betaine is classified as Generally Recognized as Safe (GRAS), while in Europe, the European Commission permits its use in foods at levels of at least 500 mg per serving due to its role in supporting the methionine cycle [129]. Betaine and its derivatives exert antimicrobial effects primarily by disrupting microbial membranes, thereby inhibiting growth and preventing biofilm formation [130]. These compounds are microbicidal and degrade into non-toxic byproducts [131]. Also, polycaprolactam- and polyhexamide-based betaine derivatives display broad bacteriostatic and fungistatic activity, reducing biofilm formation by *Staphylococcus aureus*, *Enterococcus faecalis*, *Escherichia coli*, *Pseudomonas aeruginosa*, *Klebsiella pneumoniae*, and various fungal species [132]. Their minimum inhibitory concentrations typically range from 0.5 to 8 mg/L [133]. Betaine esters demonstrate antimicrobial efficacy comparable to that of conventional disinfectants, making them suitable for food-related and topical applications [134]. Their activity is influenced by alkyl chain length, with shorter-chain derivatives performing better in protein-rich or low-temperature environments [135]. Betaine-modified polymers exhibit enhanced interactions with microbial surfaces [135]. Betainated chitosan derivatives remain soluble and retain antimicrobial activity at physiological pH, outperforming native chitosan [136]. Likewise, betaine-substituted synthetic polymers show increased antibacterial potency, mainly attributable to their higher cationic charge and stronger electrostatic interactions with microbial membranes [135].

Betaine is generally well tolerated; however, several adverse effects have been documented. The most frequently reported reactions include gastrointestinal disturbances (abdominal discomfort, diarrhea, and nausea) [137].

##### Natural Essential Oils

**Figure 10:**
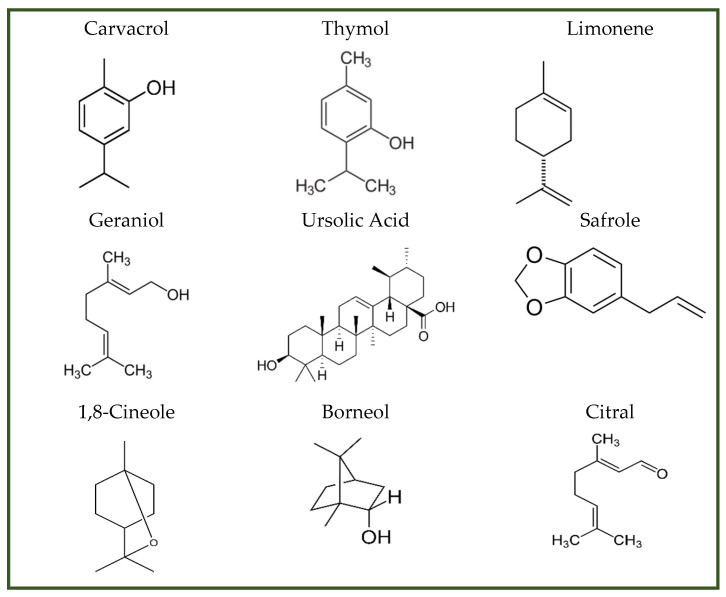
Terpenes and terpenoids with antimicrobial activity.

Natural essential oils (NEOs) are volatile plant-derived compounds obtained from various tissues, including fruits, seeds, flowers, leaves, bark, and roots. They are complex mixtures of both polar and nonpolar constituents, typically comprising 20–60 individual compounds at varying concentrations. NEOs are generally characterized by the presence of two or three principal components occurring at relatively high levels (20–70%), while the remaining constituents are present only in trace amounts [138]. Components of essential oils can be broadly classified into two groups with distinct biosynthetic origins: terpenes (primarily monoterpenes and sesquiterpenes) and terpenoids (mainly monoterpenoids). Monoterpenes (C_10_), formed by the condensation of two isoprene units (C_5_), are the most abundant constituents in many essential oils and may account for up to 90% of their composition. Terpenes and terpenoids are lipophilic molecules that exhibit antibacterial activity against both Gram-positive and Gram-negative bacteria [138]. Gram-negative bacteria are generally more resistant to natural essential oils (NEOs) than Gram-positive bacteria due to differences in cell wall architecture. While Gram-positive bacteria possess a thick peptidoglycan layer, Gram-negative bacteria have a thinner peptidoglycan layer and an additional outer phospholipid membrane that restricts the penetration of essential-oil constituents [139]. Their potency is strongly influenced by the nature and position of functional groups, such as aldehydes, ketones, acetate moieties, and hydroxyl groups, as well as by their stereochemistry. In general, β-isomers display greater antimicrobial activity than their α-isomer counterparts, and trans isomers tend to be more potent than cis forms (Figure 10) [140]. Oxygenated terpenes also exhibit greater antibacterial activity than hydrocarbon terpenes [141] (Table 3).

The terpenoids interact directly with bacterial membranes and intracellular metabolic systems, contributing to their broad-spectrum antimicrobial activity. Their primary mechanisms of action include membrane disruption, interference with energy metabolism, quorum-sensing inhibition, respiration suppression, and efflux pump inhibition [142]. They also reduce intracellular ATP levels and inhibit H+ ATPase activity, resulting in intracellular acidification and disruption of energy homeostasis [143]. By blocking acyl-homoserine lactone signaling, terpenoids interfere with quorum sensing, thereby diminishing bacterial communication, attenuating virulence, and limiting the development of resistance [144]. In addition, they impair respiratory processes by reducing oxygen uptake and disrupting oxidative phosphorylation [145]. Terpenoids can also inhibit efflux pumps, preventing bacteria from expelling antimicrobial agents [146].

Essential oils can be incorporated into food active packaging systems to maintain freshness, safety, and quality. Their antimicrobial efficacy, however, is reduced in foods with low water activity and is further influenced by factors such as fat, carbohydrate, protein, and salt content, as well as pH, storage temperature, and the oxygen concentration within the package.

Several factors may constrain the antimicrobial use of essential oils. Plant extracts and natural aromas can be expensive, sometimes costing more than the synthetic alternatives they are intended to replace. Moreover, although terpenes are generally regarded as safe at low concentrations, they can exhibit dose-dependent adverse effects, as exemplified by carvacrol and eugenol.

Carvacrol is an FDA-approved food preservative [147] that, at higher doses, has been associated with alterations in hematological and biochemical parameters (e.g., reduced hemoglobin and hematocrit, increased CPK, triglycerides, and LDH) and with pancreatic stress, including elevated α-amylase activity [148].

Eugenol can display neurotoxic and hepatotoxic properties, as its metabolism generates reactive quinone intermediates that induce oxidative stress and mitochondrial dysfunction, and prolonged intake has been linked to irreversible neurological and liver damage [149].

Similarly, terpenoids such as ursolic acid (UA) have shown dose-related adverse effects in animal studies, including behavioral alterations, depression-like symptoms, reductions in white blood cells and platelet counts, and histopathological changes in liver, kidney, and spleen tissues, together with mild hepatic and renal degeneration following repeated oral administration [150].

Biotechnological approaches, such as enzymatic or microbial fermentation and the use of recombinant microorganisms, offer promising strategies to produce these compounds more efficiently, sustainably, and at lower cost [151].

**Table 3:** Antimicrobial mechanisms of representative terpenes and related compounds, their primary microbial targets, and observed biological effects.

Mechanism	Representative Compounds	Target Microorganism	Effect	Reference
Membrane disruption	1,8-cineole, carvacrol, thymol, limonene	*Escherichia coli*, MRSA, *Staphylococcus aureus*	Leakage, bacteriostatic	[152]
ATP inhibition	Eugenol, thymol, carvacrol	*Escherichia coli*	Acidification, ATP loss	[153]
Quorum sensing/biofilm	Carvacrol, thymol, borneol, citral	*Listeria*, *Pseudomonas*	Biofilm prevention	[154]
Respiration inhibition	Monoterpenes	Aerobic bacteria	Reduced oxygen uptake, uncoupled phosphorylation	[155]
Efflux pump inhibition	Geraniol, thymol, ursolic acid, safrole	*Enterobacter*, *Escherichia coli*, *Staphylococcus. aureus*	Resistance suppression	[156]

##### Alkaloids

Alkaloids are basic, nitrogen-containing organic compounds that form a highly heterogeneous class of secondary metabolites. They can be categorized by chemical structure into several major groups, including isoquinolines, pyrroles, pyridines, quinolines, indoles, and related subfamilies (Table 4).

Numerous in vivo and clinical studies have demonstrated that alkaloids possess a wide range of pharmacological activities, including anticancer, anti-inflammatory, antiviral, and antibacterial effects [157]. Investigations into their antibacterial mechanisms reveal that natural alkaloids can compromise bacterial membrane integrity, interfere with DNA function [158], and inhibit protein synthesis, showing efficacy even against resistant pathogens such as methicillin-resistant *Staphylococcus aureus* [159].

Isoquinoline alkaloids, including tc [160], chelerythrine [114], and sanguinarine [161], exhibit potent activity against *Bacillus subtilis*, *Staphylococcus aureus*, *Pseudomonas aeruginosa*, and *Candida albicans*, with minimum inhibitory concentrations (MICs) often comparable to those of standard antibiotics.

Pyridine alkaloids such as caranine [162] and quinolizidine derivatives from *Zingiberis* [163] and *Sophora tonkinensis* [164] demonstrate antibacterial and antifungal effects across Gram-positive and Gram-negative pathogens.

Indole alkaloids, ranging from strychnine derivatives [165] to bis-indolic compounds such as dionemycin, show remarkable efficacy against resistant strains, including MRSA [166]. Steroidal alkaloids isolated from *Holarrhena* and *Combretum* species display moderate but clinically relevant activity [167].

**Figure 11:**
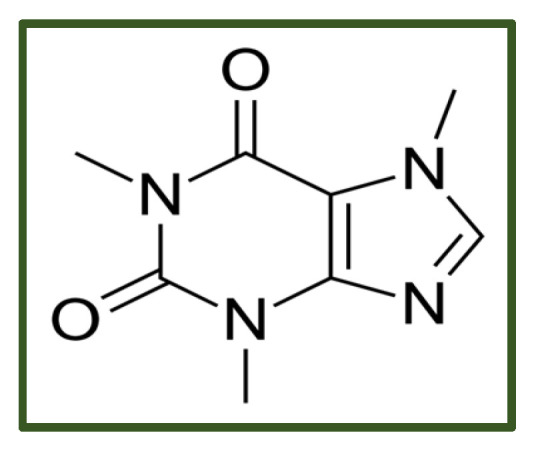
Caffeine.

Caffeine (Figure 11) is a multifunctional antibacterial plant-derived methylxanthine. Its planar, amphiphilic structure facilitates membrane insertion [168], disruption of hydrophobic packing, increased permeability, and ion leakage through mechanosensitive channels [166]. Caffeine also intercalates into DNA, impairing replication and repair—an effect supported by whole-genome sequencing of *Staphylococcus aureus*, which revealed reduced read mapping, lower coverage, and altered GC content following exposure. Computational studies further suggest that caffeine can bind LtaS, an enzyme involved in lipoteichoic acid synthesis, indicating potential interference with cell wall biogenesis [169].

Beyond *Stafilococcus aureus*, caffeine inhibits biofilms and alters membrane fluidity in *Pseudomonas* and reduces adhesion and biofilm development in *Listeria* [170,171]. It also functions as an antibiotic adjuvant, lowering MICs of several drugs against major Gram-negative pathogens, including *Pseudomonas aeruginosa*, *Acinetobacter baumannii*, and *Klebsiella pneumoniae* [172]. It also exhibits potent antibiofilm and anti-persister activity, effectively disrupting biofilms and eradicating dormant cells in *Listeria monocytogenes*, *Staphylococcus aureus*, *Escherichia coli*, and *Pseudomonas aeruginosa* [173].

Some adverse effects associated with the use of alkaloids have been reported. For example, berberine consumption is commonly linked to gastrointestinal disturbances, including constipation and diarrhea [174], and high caffeine intake may elicit undesirable effects such as anxiety, restlessness, insomnia, and psychomotor agitation [172].

##### Saponins

**Figure 12:**
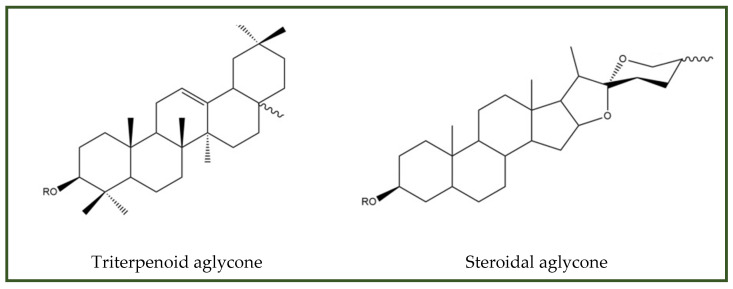
Saponin aglycones.

Saponins are glycosides composed of a triterpenoid or steroidal aglycone linked to one or more carbohydrate moieties (Figure 12) [175].

Experimental evidence demonstrates that saponins destabilize microbial membranes, increasing permeability and ultimately leading to cell death. In addition, saponins exhibit synergistic activity with conventional antibiotics by enhancing drug uptake and inhibiting biofilm formation [176,177,178,179].

Saponins’ antimicrobial applications are constrained by pronounced cytotoxicity, particularly strong hemolytic effects. Their interaction with erythrocyte membranes disrupts the lipid bilayer and promotes hemoglobin release, with toxicity strongly dependent on structural features such as aglycone type, glycosylation pattern, and presence of specific functional groups [180].

##### Cysteine Sulfoxides

**Figure 13:**
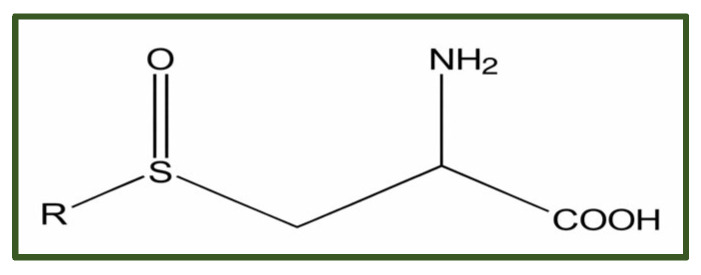
Cysteine sulfoxide aglycones.

Cysteine sulfoxides are sulfur-containing amino acid derivatives found in *Allium* species (garlic, onion, leek, chives) (Figure 13). They serve as precursors to bioactive organosulfur compounds such as allicin, ajoene, and diallyl sulfides, which exhibit antimicrobial, antioxidant, and broader therapeutic properties [181]. In vitro assays have demonstrated that ethanolic extracts rich in these compounds inhibit biofilm formation by a range of clinically relevant pathogens, including *Escherichia coli*, *Pseudomonas aeruginosa*, *Bacillus subtilis*, *Staphylococcus aureus*, and *Candida albicans.* These effects are attributed mainly to the ability of organosulfur molecules to disrupt quorum sensing, impair cell–cell communication, and interfere with the structural integrity of developing biofilms [182].

Cysteine sulfoxides are associated with several adverse effects. For example, allicin’s pungent and irritating odor can trigger allergic reactions, and cases of dermatitis, heartburn, asthma, urticaria, edema, gastrointestinal disturbances such as nausea, vomiting, diarrhea, and flatulence have been documented. To mitigate these limitations, a variety of advanced delivery systems have been developed to improve allicin’s stability, loading capacity, and bioavailability, including nanoparticles, gels, liposomes, and micellar formulations [183].

##### Isothiocyanate

**Figure 14:**
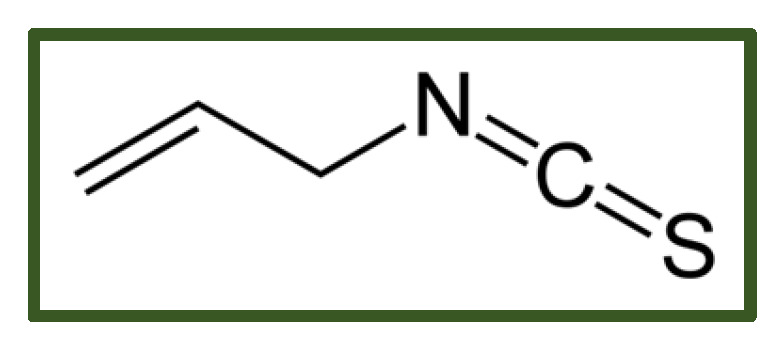
Allyl isothiocyanate.

Allyl isothiocyanate (Figure 14) has broad-spectrum antimicrobial activity against *Listeria monocytogenes*, *Bacillus cereus*, *Campylobacter* spp., *Escherichia coli*, *Staphylococcus aureus*, *Salmonella* spp., as well as fungi and yeasts [184,185]. Its mechanism of action involves disruption of the cellular membrane potential, inhibition of aminopeptidase activity, and suppression of dehydrogenase reductase-1, with these effects becoming detectable within 48 h of microbial growth [186,187]. Evidence also supports synergistic effects when allyl isothiocyanate is combined with other antimicrobials [188].

Allyl isothiocyanate can induce, in a dose-dependent manner, mucosal irritation, cytotoxicity, and organ damage, and can cause developmental and reproductive toxicity, especially via oral administration [189].

##### Lectins

**Figure 15:**
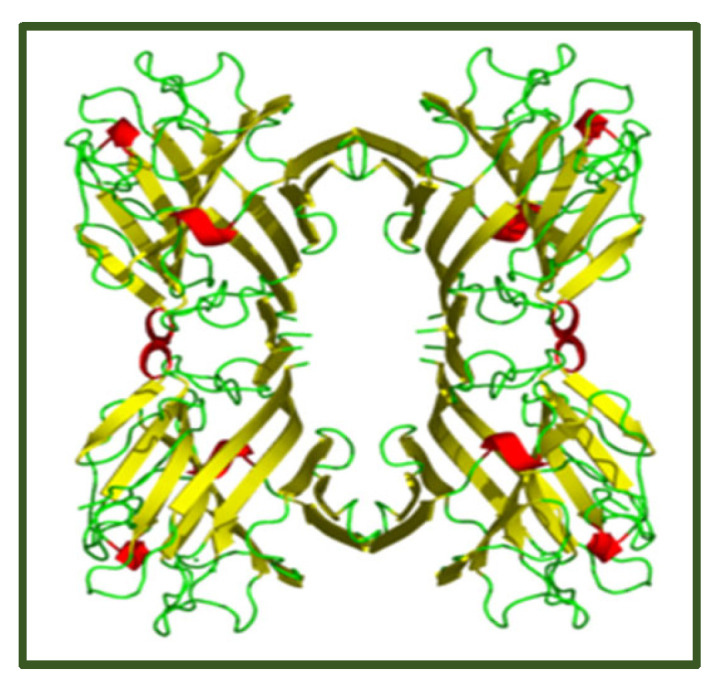
Lectin.

Lectins are carbohydrate-binding proteins that function independently of the immune system. They contain at least one carbohydrate-recognition domain, enabling specific and reversible interactions with microbial glycans [190] (Figure 15).

Lectins exert antimicrobial activity by agglutinating bacterial cells, disrupting membrane integrity, and inhibiting biofilm formation. These effects arise from their ability to recognize and bind specific cell wall components, such as lipopolysaccharides and peptidoglycans, thereby interfering with essential structural and signaling processes [191]. Recent studies show that lectins can be used as natural agents to prevent biofilms and as adjuncts to antibiotic treatments, especially against bacteria that are resistant to many drugs [192]. Napoleão et al. found that lectins can disrupt biofilm structures and enhance the efficacy of antibiotics [193]. Souza et al. studied plant lectins with antibacterial and antibiofilm effects, demonstrating their activity against both Gram-positive and Gram-negative bacteria [194]. Belfiori et al. reported that *Vicia ervilia* lectin inhibited biofilm growth in both Gram-positive and Gram-negative bacteria, as well as in *Candida albicans*, suggesting it could be helpful in therapy [195]. Carneiro et al. showed that marine lectins enhanced the effectiveness of antibiotics against harmful bacteria [196]. Andrade et al. described Afil, a lectin from *Aplysina fistularis*, that inhibited biofilm growth and synergized with antibiotics against resistant strains [197].

Lectins’ practical application is limited by concerns related to toxicity. Some dietary lectins may exert toxic effects at high doses or under specific conditions, potentially triggering immune activation, allergic reactions, or autoimmune-like responses. Interindividual variability and optimal dosing remain key challenges [198].

##### Polypeptides (AMPs)

AMPs are short polypeptides, typically composed of 12–50 amino acids, and are conserved across all forms of life. In humans, they are produced primarily by epithelial cells and phagocytes as components of the innate immune system, and are widely distributed in tissues and on mucosal surfaces [199]. AMPs exhibit activity against a broad spectrum of bacteria, fungi, protozoa, and viruses. In contrast to many conventional antibiotics, which typically act on specific cellular targets, AMPs employ multiple complementary mechanisms, a feature that helps slow the emergence of resistance. They are generally less toxic because they are ultimately degraded into amino acids rather than harmful byproducts. Their relatively short sequences also facilitate cost-effective synthesis, and unlike many traditional antibiotics, they typically exert minimal disruption on the host microbiota [200]. Bioactive peptides can fight bacteria like *Staphylococcus aureus*, *Propionibacterium acnes*, *Pseudomonas aeruginosa*, *Enterococcus faecium*, *Acinetobacter baumannii*, *Klebsiella pneumoniae*, and *Enterobacter* species. Because of these properties, AMPs are considered promising candidates for incorporation into food supplements and cosmeceutical formulations. Their antimicrobial activity arises primarily from their ability to form pores or channels in microbial membranes, leading to cytoplasmic leakage and cell death. In addition, many AMPs inhibit essential cellular processes, including cell division, protein folding, cell wall and protein biosynthesis, nucleic acid synthesis, and lipopolysaccharide formation [201]. Host defense peptides exhibit modes of action comparable to those of certain membrane-targeting antibiotics, including daptomycin and colistin. Moreover, interactions between antimicrobial agents and the host immune system can modulate cytokine responses and influence the expression of microbial virulence factors [202]. However, AMPs face significant challenges in clinical application. They are often structurally unstable and readily degraded by proteolytic enzymes, which substantially reduces their bioavailability and therapeutic effectiveness in vivo [203]. Other limitations include poor bioavailability [204] and short plasma half-lives [205]. Moreover, AMPs can exhibit toxicity toward mammalian cells, as their hydrophobicity, net positive charge, and secondary structure may promote interactions with zwitterionic membranes, leading to hemolysis or cytotoxicity despite the natural selectivity conferred by microbial membrane composition. Structural optimization of AMPs, through targeted modifications of amino acid residues and physicochemical parameters, remains essential to enhance antimicrobial potency while minimizing toxicity and improving their suitability for clinical development [206].

#### 5.2.3. Emerging Technologies

##### Nanotechnology

**Figure 16:**
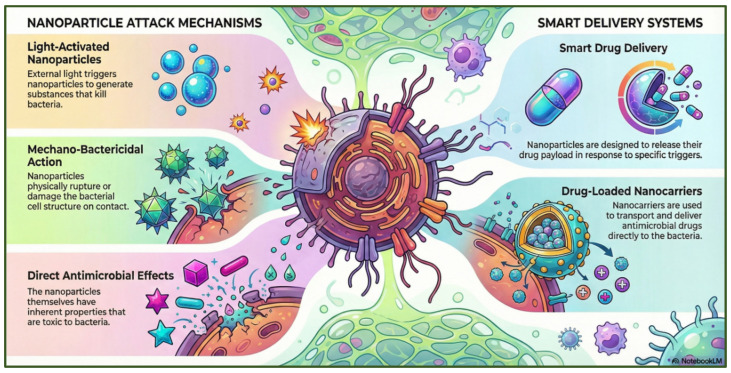
Nanoparticle-based antimicrobial strategies, including direct bactericidal actions and smart delivery systems for targeted drug release. The figure was generated using NotebookLM (Gemini 1.5 model), consulted in February 2026.

Nanotechnology enhances drug delivery, disrupts biofilms, and supports the development of novel antimicrobial agents. Due to their small size, nanomaterials can alter the absorption, distribution, and clearance of therapeutic compounds, thereby improving overall treatment efficacy [207]. Current research is focused on engineering nanoparticles (metal- or polymer-based nanoparticles) capable of detecting antibiotic-resistant bacteria and potentiating the activity of conventional antibiotics. Metal-based nanoparticles, such as silver, zinc oxide, and gold, possess intrinsic antimicrobial properties [208]. Their bactericidal activity is attributed to several mechanisms, including the generation of reactive oxygen species, inhibition of protein synthesis, and direct damage to bacterial DNA [209]. Polymer-based nanomaterials, such as dendrimers and nanogels, and carbon-based nanostructures, such as graphene, carbon nanotubes, fullerenes, carbon dots, and nanodiamonds, can act either by direct contact (hydrophobic, electrostatic, chelation) or by releasing agents that penetrate cell walls [210]. By serving as both active antimicrobial agents and delivery platforms, these nanotechnologies represent versatile tools in the fight against antimicrobial resistance [211] (Figure 16).

Several challenges remain, including concerns related to toxicity [212], scalability, and regulatory approval, all of which must be resolved before complete clinical translation can be achieved [213]. For example, TiO_2_ nanoparticles have shown contradictory cytotoxicity outcomes across studies [214,215], and gold nanomaterials have similarly been reported to exhibit both toxic and non-toxic effects [216]. Green synthesis approaches, such as those using plant extracts, help reduce toxicity while providing cost-effective, environmentally sustainable routes to nanoparticle production [217,218].

##### Monoclonal Antibodies

Antibacterial monoclonal antibodies (mAbs) bind bacterial epitopes with high specificity, recruit host immune mechanisms, and generally exhibit favorable safety profiles. Advances in modern technologies have facilitated the identification of mAbs that target a wide range of bacterial structures, including those that do not require penetration of the cell envelope to exert their effects [219].

Three monoclonal antibodies, obiltoxaximab, raxibacumab, and bezlotoxumab, have received regulatory approval, all of which target virulence factors of Gram-positive pathogens, specifically the *Bacillus anthracis* protective antigen or *Clostridium difficile* toxin B. An antibody directed against *Staphylococcus aureus* α-toxin did not demonstrate efficacy in pneumonia clinical trials, although additional candidates remain under investigation. For Gram-negative bacteria, promising approaches include mAbs targeting Shiga toxin–producing *Escherichia coli* (Stx1/Stx2), which have shown safety in humans and protective effects in murine models, as well as antibodies that inhibit the type III secretion system; however, their clinical utility has yet to be established [220]. Despite these advantages, their clinical success has remained limited due to inconsistent clinical efficacy. This variability is influenced by pathogen heterogeneity, complex host–pathogen interactions, biofilm formation, and substantial differences in patient immune status. Additional constraints arise from suboptimal trial designs, inadequate patient stratification, and the limited use of rapid diagnostic tools that ensure timely and accurate pathogen identification. To enhance the translational impact of future studies, more rigorous and stratified clinical trial designs are required, together with early mAb administration and standardized antibiotic co-administration—areas that remain insufficiently explored. Furthermore, optimized dosing strategies and rational mAb combination regimens represent promising yet largely uncharted avenues. Finally, high production costs, manufacturing complexity, and regulatory hurdles continue to pose significant barriers to broader implementation [221].

## 6. Limitations of This Work and Future Perspectives

Despite providing an integrated overview of antimicrobial resistance mechanisms and emerging therapeutic strategies, this review presents several limitations that should be acknowledged to contextualize the current state of knowledge. Evidence on antimicrobial contamination and resistance patterns remains uneven across geographical regions and environmental matrices, limiting the ability to draw globally representative conclusions. Most available data focus predominantly on bacterial resistance, whereas fungal, viral, and parasitic resistance are comparatively underexplored, restricting a comprehensive assessment of cross-kingdom antimicrobial challenges. Several critical gaps further constrain the application of emerging antimicrobial therapies. For many innovative approaches, such as antimicrobial peptides, antisense technologies, nanomaterials, bacteriophage-based interventions, and monoclonal antibodies, information on clinical readiness, regulatory barriers, long-term safety, and cost-effectiveness remains limited or fragmented. These constraints hinder translation from experimental models to real-world clinical or environmental settings. Additionally, given the field’s rapid evolution, their potential ecological or evolutionary impacts may not yet be fully captured in the available literature.

Future research should prioritize harmonized global surveillance systems, standardized methodologies for assessing antimicrobial resistance across environmental compartments, and expanded investigation of non-bacterial pathogens. For emerging therapies, rigorous preclinical and clinical studies are needed to evaluate pharmacokinetics, toxicity, scalability, and regulatory compliance. Integrating interdisciplinary approaches, spanning microbiology, materials science, pharmacology, environmental science, and public health, will be essential to accelerate the development of safe, effective, and sustainable antimicrobial alternatives. Continued innovation, coupled with coordinated international efforts, will be crucial to mitigate the growing burden of antimicrobial resistance and to guide the next generation of therapeutic strategies.

## 7. Conclusions

The widespread use of antibiotics has accelerated the emergence of antibiotic-resistant pathogens, which are now increasingly detected in both animal- and plant-derived foods. Contaminated food represents a major transmission route, amplifying risks to public health. Effective mitigation requires strengthened global surveillance systems, more rigorous regulatory frameworks, and the development of innovative alternatives. Although antimicrobial peptides, natural bioactive compounds, nanomaterials, and antibody-based therapies show considerable promise against multidrug-resistant pathogens, challenges related to toxicity, scalability, and clinical translation remain substantial. A coordinated, multidisciplinary approach, integrating microbiology, food science, pharmacology, and public health, is thus essential to curb antimicrobial resistance and safeguard both food safety and human health.

## Figures and Tables

**Table 1:** Alternative antimicrobial therapies.

Approach	Principle	Example	Limitations	References
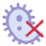 Antivirulence	Block virulence factors	Sibofimloc (*Escherichia coli* FimH); Bezlotoxumab (*Clostridium difficile*)	The compound shows no intrinsic microbicidal activity, and clinical trial evidence is currently absent. Its activity appears contingent upon host immune mechanisms or synergistic co-therapies.	[7,81]
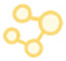 Adjuvants	Sensitize bacteria to drugs	SPR741 (membrane disruptor); Vaborbactam (β-lactamase inhibitor)	Potential toxicity and the incomplete identification of compounds or substances with the essential physicochemical characteristics required for efficacy.	[82,83]
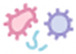 Consortia	Restore microbiota balance	VE303 (Clostridia strains); Rebyota (fecal microbiota transplantation for *Clostridium difficile*)	Potential transmission of resistant strains and reduced efficacy relative to full fecal microbiota transplantation.	[84,85]
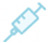 Vaccines	Induce protective immunity	PCV20 (*Staphylococcus pneumoniae* polysaccharides)	Complexity of antigen selection and limited evidence regarding long-term immune durability.	[86,87]

**Table 2:** Antimicrobial Activity of Phenols and Polyphenols.

Phenol/Polyphenol	Target Microorganism	Mechanism of Action	References
Phenolic acid			
Salicylic acid 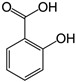	*Escherichia coli* and *Staphylococcus aureus*	Disrupt bacterial cell walls and cell membranes	[108]
Gallic acid 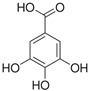	*Pseudomonas aeruginosa*, *Staphylococcus aureus*, *Escherichia coli*, *Klebsiella pneumonia*, *Listeria monocytogenes*, *Chromobacterium violaceum*, and *Campylobacter jejuni*	Damage cell walls	[109]
p-Coumaric acid 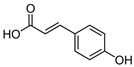	Gram-positive pathogenic bacteria (*Staphylococcus aureus*, *Listeria*), Gram-negative pathogenic bacteria (*Escherichia coli*, *Salmonella*), and plant pathogenic fungi (*B. cinerea*, *P. expansum*, and *A. alternata*)	Disruption of the bacterial membranes, binding of DNA, blocking vital functions, and causing cell death	[110,111]
Simple phenols			
Tyrosol 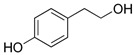	*Escherichia coli*, *S. aureus*, *Candida parapsilosis*, *Candida albicans, Candida glabrata*, and *Streptococcus mutans*	Quorum sensing and biofilm inhibition	[112,113]
Bromophenol 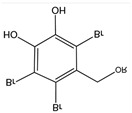	*Candida albicans*, *Pseudomonas fluorescence*, and *Staphylococcus aureus*	Damage membranes	[114]
Polyphenols			
Chalcones 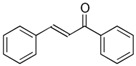	*Staphylococcus aureus*	Damage the membranes	[115]
Anthocyanins 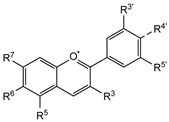	*Staphylococcus aureus*	Damage of the bacterial cells by disrupting the wall, membrane, and matrix integrity, inhibiting enzymes, blocking biofilms, and impairing metabolism and growth	[116]
Catechin 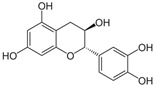	*Bacillus cereus*, *Escherichia coli*, *Campylobacter jejuni*, *Helicobacter pylori*, *Clostridium perfringens*, *Staphylococcus aureus*, *Legionella pneumophila*, *Pseudomonas aeruginosa*, *Shigella flexneri*, and *Vibrio cholerae*	Inhibition of cell membrane, cell wall, nucleic acid, and protein synthesis, while also blocking key metabolic pathways—including virulence factors, toxin production, iron chelation, and oxidative stress responses	[117]
Curcumin 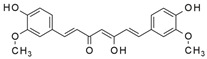	Gram-positive and Gram-negative *Staphylococcus aureus*, *Salmonella paratyphi*, and *Bacillus subtilis*	Damages bacterial cell walls and membranes, interferes with DNA and protein functions, and inhibits quorum sensing	[118]

**Table 4:** Classification of alkaloid subgroups.

Subgroup	Notes
Isoquinolines (e.g., berberine).	Based on the isoquinoline skeleton 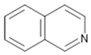
Quinolines (e.g., dictamnine, skimmianine, furoquinoline).	Based on the quinoline ring system 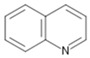
Pyridines (e.g., metanicotine, niphatesines, cribrochalinamines, manzamine A, haliclamine A, 3-alkylpyridines)	It contains a six-membered aromatic ring with one nitrogen atom 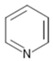
Pyrroles (e.g., niphatesines, cribrochalinamines, and 3-alkylpyridine)	Based on a five-membered heterocyclic ring with one nitrogen atom 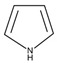
Indoles (e.g., voacafricines, gelselegandines)	Based on a fused benzene and pyrrole ring system 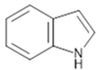

## Data Availability

No new data were created or analyzed in this study. Data sharing is not applicable to this article.
